# Supplementary material for: Structural basis of Cdk7 activation by dual T-loop phosphorylation
Source: Nat Commun. 2024 Aug 3;15:6597. doi: 10.1038/s41467-024-50891-z (PMC11297931; doi:10.1038/s41467-024-50891-z)
Supplement: Supplementary file 1 — Supplementary Information [file 41467_2024_50891_MOESM1_ESM.pdf]

Supplementary Information

Structural basis of Cdk7 activation by dual T-loop phosphorylation

Robert Düster[1,2], Kanchan Anand[1], Sophie C. Binder[1], Maximilian Schmitz[1], Karl Gatterdam[1], Robert P. Fisher[2]* & Matthias Geyer[1]*

[1] Institute of Structural Biology, University of Bonn, Venusberg-Campus 1, 53127 Bonn, Germany.

[2] Department of Oncological Sciences, Icahn School of Medicine at Mount Sinai, New York, NY, USA.

* Correspondence should be addressed to: matthias.geyer@uni-bonn.de or robert.fisher@mssm.edu

The PDF file includes:

- Supplementary Figures S1 – S8
- Supplementary Table 1

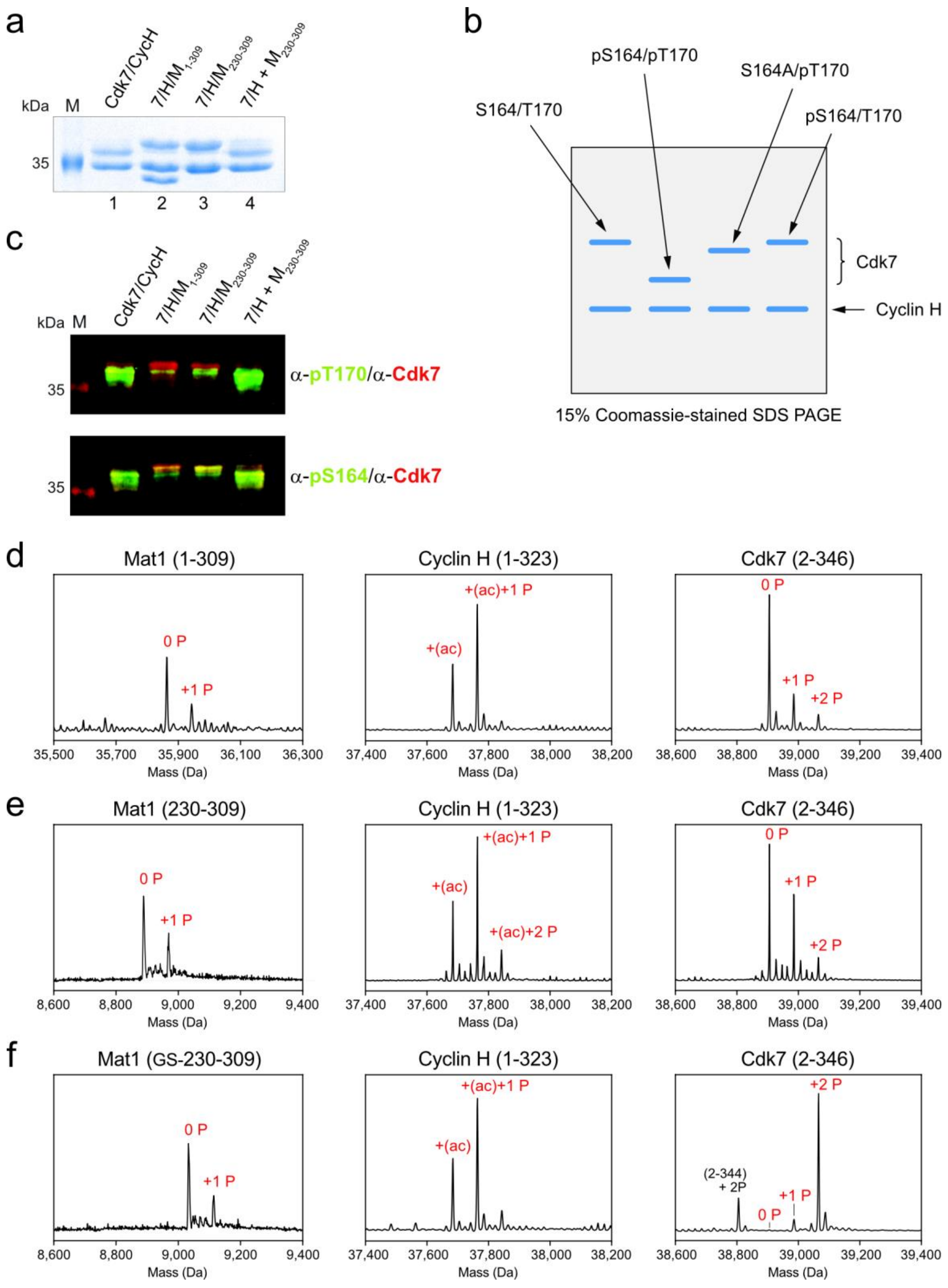

Supplementary Fig. 1 | Cdk7 T-loop phosphorylation and protein integrity. **a**, SDS-PAGE analysis of the samples shown in **Fig. 1a**. Running parameters were changed to separate phosphorylated Cdk7 from Cyclin H. 10 µl at a concentration of 2.6 µM were separated on a 15% SDS-PAGE until the 35 kDa marker band of the prestained marker had migrated ~60% through the gel. **b**, Cartoon of the running behavior of Cdk7 phospho-isoforms in SDS-PAGE. **c**, Merged images of the western blots shown in **Fig. 1a**. **d-f**, Molecular masses of intact proteins determined by ESI–(LC)–MS indicating the phosphorylation status. **d**, Protein masses from the co-expression of the full length Cdk7/Cyclin H/Mat1$_{1-309}$ complex (corresponding to lane 2 in panel **a**). **e**, Protein masses from the co-expression of truncated Cdk7/Cyclin H/Mat1$_{230-309}$ (lane 3 in panel **a**). **f**, Protein masses upon addition of Mat1$_{230-309}$ to co-expressed Cdk7/Cyclin H (lane 4 in panel **a**). Source data are provided as a Source Data file.

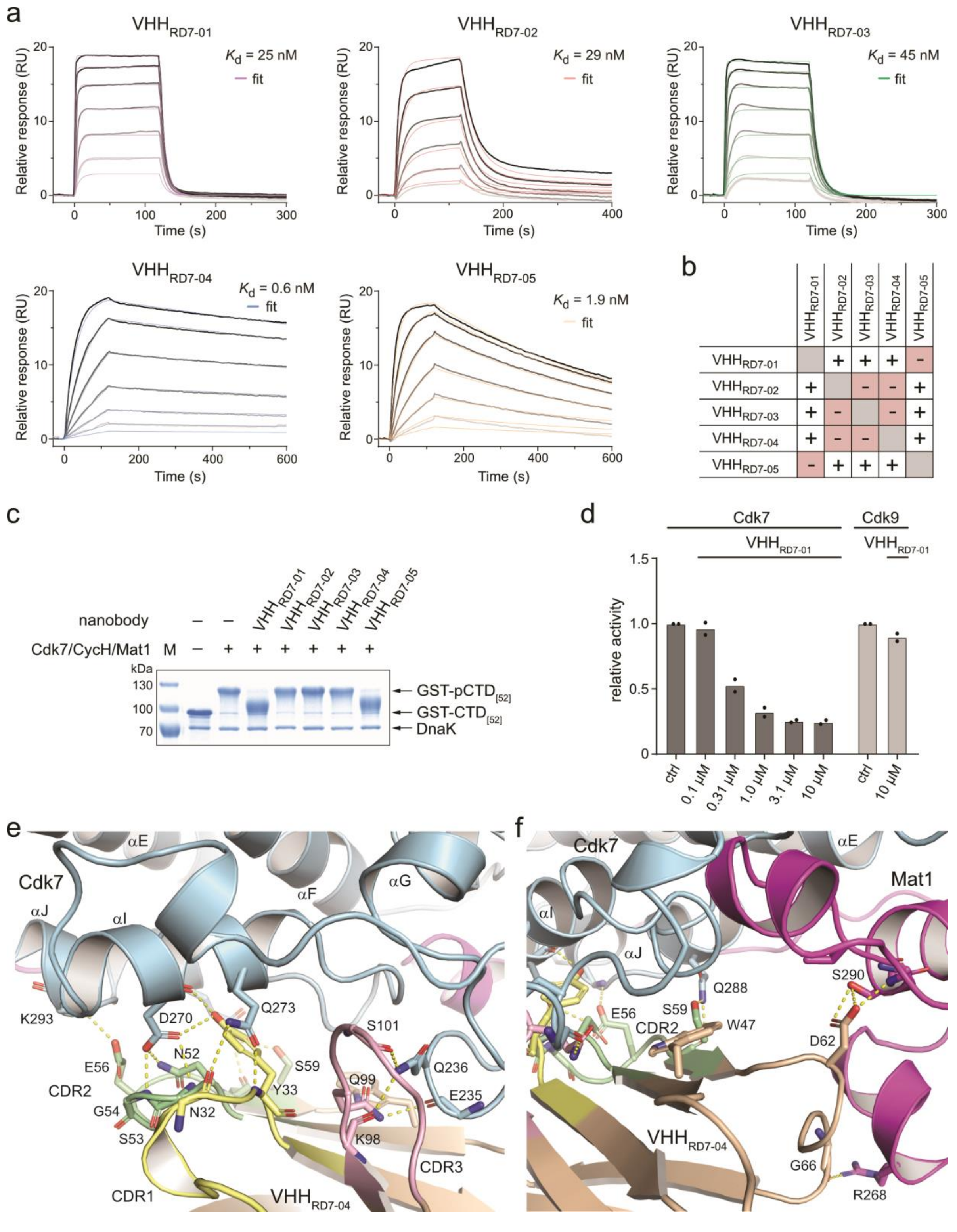

Supplementary Fig. 2 | Generation of Cdk7/Cyclin H/Mat1-binding nanobodies. **a**, SPR binding analyses of Cdk7/Cyclin H/Mat1 directed VHHs. **b**, Epitope binning of VHHs highlights two different epitopes. **c**, Impact of VHHs on Cdk7 activity. 0.1 µM Cdk7/Cyclin H/Mat1$_{230\text{-}309}$ was pre-incubated with 1 µM VHH and 10 µM GST-CTD$_{[52]}$. Reaction was started with 1 mM ATP and stopped after 15 min. Phosphorylation of the CTD was determined by SDS-PAGE analysis. **d**, Radiometric kinase assay to analyze the inhibitory potential of VHH$_{RD7\text{-}01}$. Assay was performed as in **c** but with varying concentrations of VHH$_{RD7\text{-}01}$ and with 1 mM ATP containing 0.35 µCi ^{32}[P]γ-ATP. Cdk9/CycT1$_{1\text{-}272}$ was used as a control for nanobody specificity. Data are presented as mean of duplicate measurements. **e**, **f**, Interaction networks of the VHH$_{RD7\text{-}04}$ CDRs with Cdk7/Cyclin H/Mat1$_{230\text{-}309}$. Source data are provided as a Source Data file.

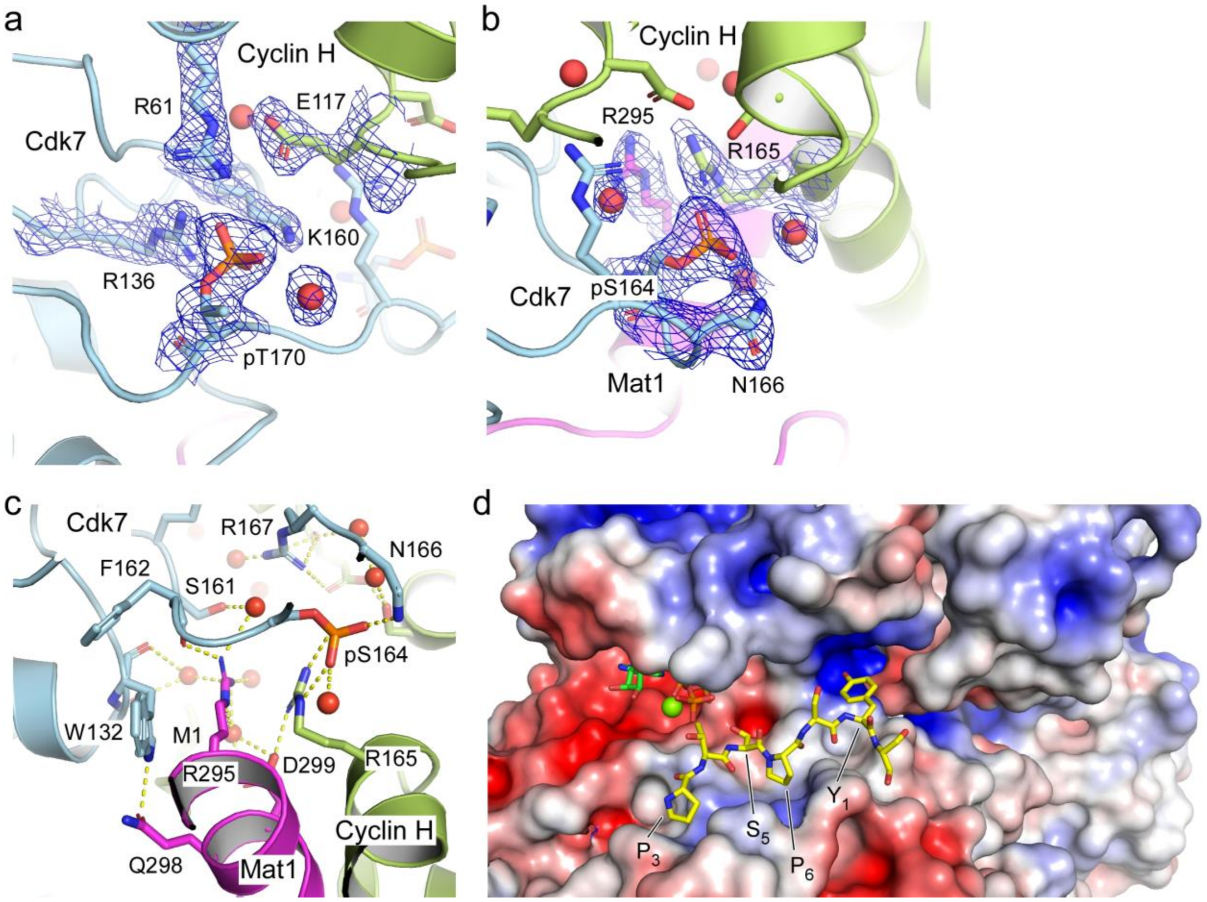

Supplementary Fig. 3 | Details of the tripartite Cdk7/Cyclin H/Mat1 complex formation. a, Electron density of pT170 and surrounding residues. The structural model is drawn in stick representation. The blue mesh is a $2mF_o\text{-}DF_c$ electron density map contoured at 1.0 σ. **b**, Representative electron density of pS164 and surrounding residues. The structural model and the electron density map are displayed as in panel **a**. **c**, Stacking interactions between R165 of Cyclin H, R295 of Mat1, and W132 of Cdk7, with pS164 of Cdk7 making salt-bridge interactions with R165 of Cyclin H. **d**, Electrostatic surface display of the Cdk7/Cyclin H/Mat1 complex structure determined here (8pyr) fused with residues 10-54, ATPγS and Mg^{2+} of Cdk7 from structure 6xbz [25] and superimposed with the substrate peptide $P_3T_4S_5P_6S_7Y_1S_2$ that was modeled on the peptide PKTPKKA from the Cdk2/CycA/substrate complex structure 3qhr [38]. The register of the PxxP binding motif in the substrate sequence aligns to S5 phosphorylation within the CTD and an $P_3xSP_6xY_1$ interaction motif.

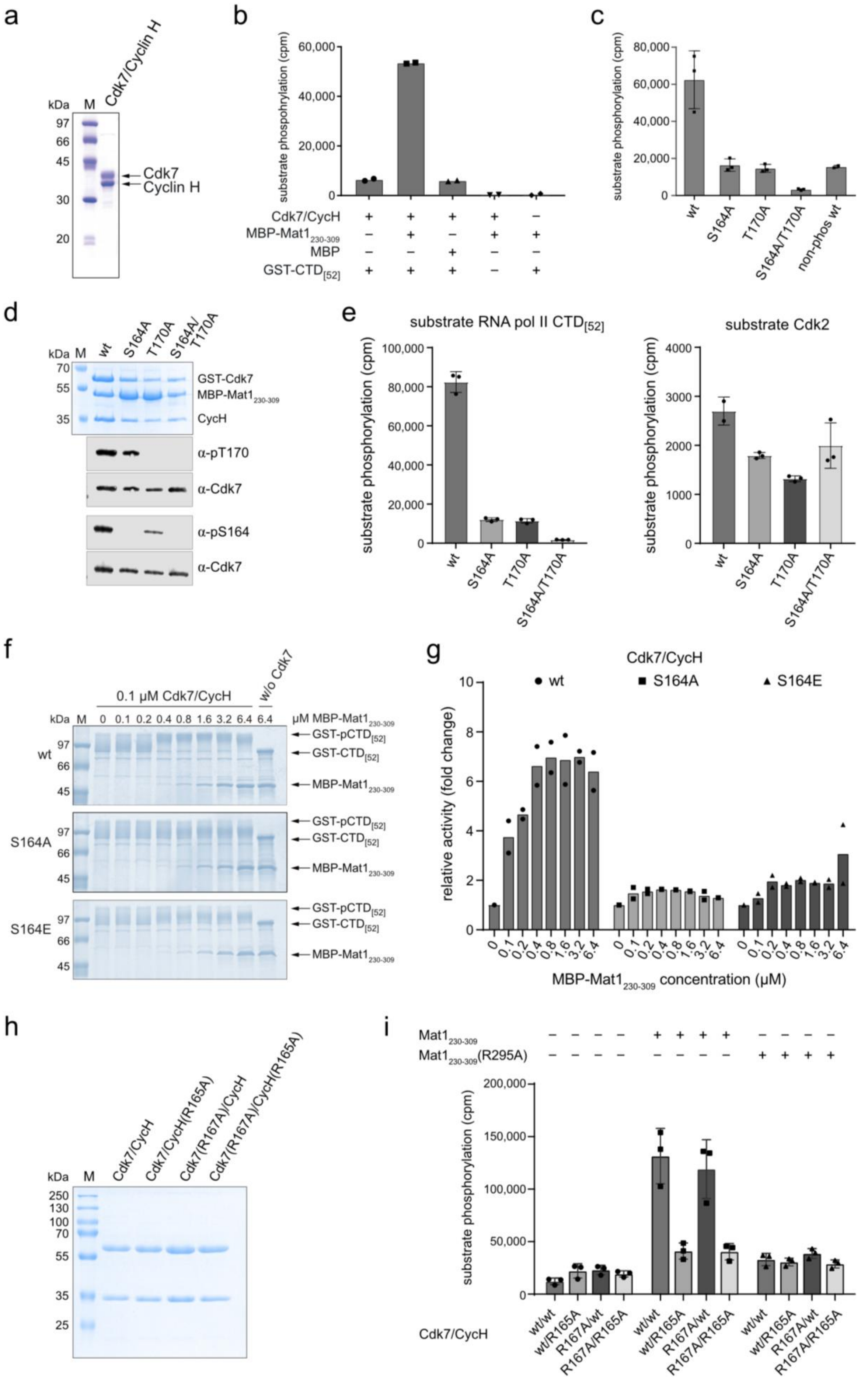

a
Cdk7/Cyclin H
kDa M
97
66
45
30
20
Cdk7
Cyclin H
b
substrate phosphorylation (cpm)
60,000
40,000
20,000
0
Cdk7/CycH + + + + −
MBP-Mat1230-309 − + − + +
MBP − − + − −
GST-CTD[52] + + + − +
c
substrate phosphorylation (cpm)
80,000
60,000
40,000
20,000
0
wt
S164A
T170A
S164A/T170A
non-phos wt
d
kDa M wt S164A T170A S164A/T170A
70
55
35
GST-Cdk7
MBP-Mat1230-309
CycH
α-pT170
α-Cdk7
α-pS164
α-Cdk7
e
substrate RNA pol II CTD[52]
substrate phosphorylation (cpm)
100,000
80,000
60,000
40,000
20,000
0
wt
S164A
T170A
S164A/T170A
substrate Cdk2
substrate phosphorylation (cpm)
4000
3000
2000
1000
0
wt
S164A
T170A
S164A/T170A
f
0.1 µM Cdk7/CycH
w/o Cdk7
kDa M 0 0.1 0.2 0.4 0.8 1.6 3.2 6.4 6.4 µM MBP-Mat1230-309
wt
S164A
S164E
97
66
45
GST-pCTD[52]
GST-CTD[52]
MBP-Mat1230-309
g
Cdk7/CycH
wt
S164A
S164E
relative activity (fold change)
10
8
6
4
2
0
0 0.1 0.2 0.4 0.8 1.6 3.2 6.4
MBP-Mat1230-309 concentration (µM)
h
kDa M
Cdk7/CycH
Cdk7/CycH(R165A)
Cdk7(R167A)/CycH
Cdk7(R167A)/CycH(R165A)
250
130
100
70
55
35
25
i
Mat1230-309 − − − − + + + + − − − −
Mat1230-309(R295A) − − − − − − − − + + + +
substrate phosphorylation (cpm)
200,000
150,000
100,000
50,000
0
Cdk7/CycH
wt/wt
wt/R165A
R167A/wt
R167A/R165A

Supplementary Fig. 4 | Establishment of ternary complex formation in vitro for kinase activity measurements. a, SDS-PAGE analysis of 3 µg Cdk7/Cyclin H complex. **b**, Radiometric kinase activity assay. 0.1 µM Cdk7/Cyclin H complex was incubated with 0.4 µM MBP or MBP-Mat1$_{230-309}$ and 10 µM GST-CTD$_{[52]}$ for 10 min prior to starting the kinase reaction by addition of 1 mM ATP containing 0.35 µCi [^{32}P]-γ-ATP. Samples were incubated for 15 min at 30°C. Data are presented as mean of duplicate measurements. **c**, Radiometric kinase activity assay. Comparison of the activities shown in Fig. 3b to non-phosphorylated Cdk7/CycH/Mat1 shown in Fig. 1a. Data were obtained within the same experiment to allow comparison of the signal strength. Data are presented as mean ± SD from triplicate measurements. **d**, SDS-PAGE and immunoblot analysis of GST-Cdk7/Cyclin H/MBP-Mat1 complexes. Protein (3 µg) was resolved on a 12% SDS gel and stained with Coomassie blue. For analysis of the Cdk7 T-loop phosphorylation status, 1 µL at 2.6 µM was immunoblotted with phospho-specific antibodies recognizing Cdk7 pT170 or Cdk7 pS164. Total Cdk7 was used as loading control. **e**, Radiometric kinase assay probing the preparations shown in **c** for activity towards RNAPII CTD and GST-Cdk2. Each Cdk7 complex at 0.1 µM was incubated with 10 µM GST-CTD$_{[52]}$ or 15 µM GST-Cdk2 in the presence of 1 mM ATP containing 0.35 µCi [^{32}P]-γ-ATP for 15 min (CTD) or 30 min (Cdk2) at 30°C. Data are presented as mean ± SD of triplicate measurements. **f**, SDS-PAGE analysis of a kinase assay titrating MBP-Mat1$_{230-309}$. Cdk7/Cyclin H complex (0.1 µM)—wild-type or S164 mutant variants, as indicated—was incubated with increasing concentrations of MBP-Mat1$_{230-309}$. Samples were incubated in the presence of 10 µM GST-CTD$_{[52]}$ for 10 min prior to starting the assay with 1 mM ATP. After 15 min at 30°C, assay was stopped with 2xSDS sample buffer. **g**, Radiometric kinase assay. As in panel **f**, but with addition of 0.35 µCi [^{32}P]-γ-ATP. Phosphorylation was measured by liquid scintillation counting. Data are presented as mean of duplicate measurement. **h**, SDS-PAGE analysis of the GST-Cdk7/Cyclin H complexes used in **Fig. 3f**, 2 µg each. **i**, Absolute counts of one experiment of the data shown in **Fig. 3f**, highlighting similar activity of the binary preparations. Data are presented as mean ± SD of triplicate measurements. Source data are provided as a Source Data file.

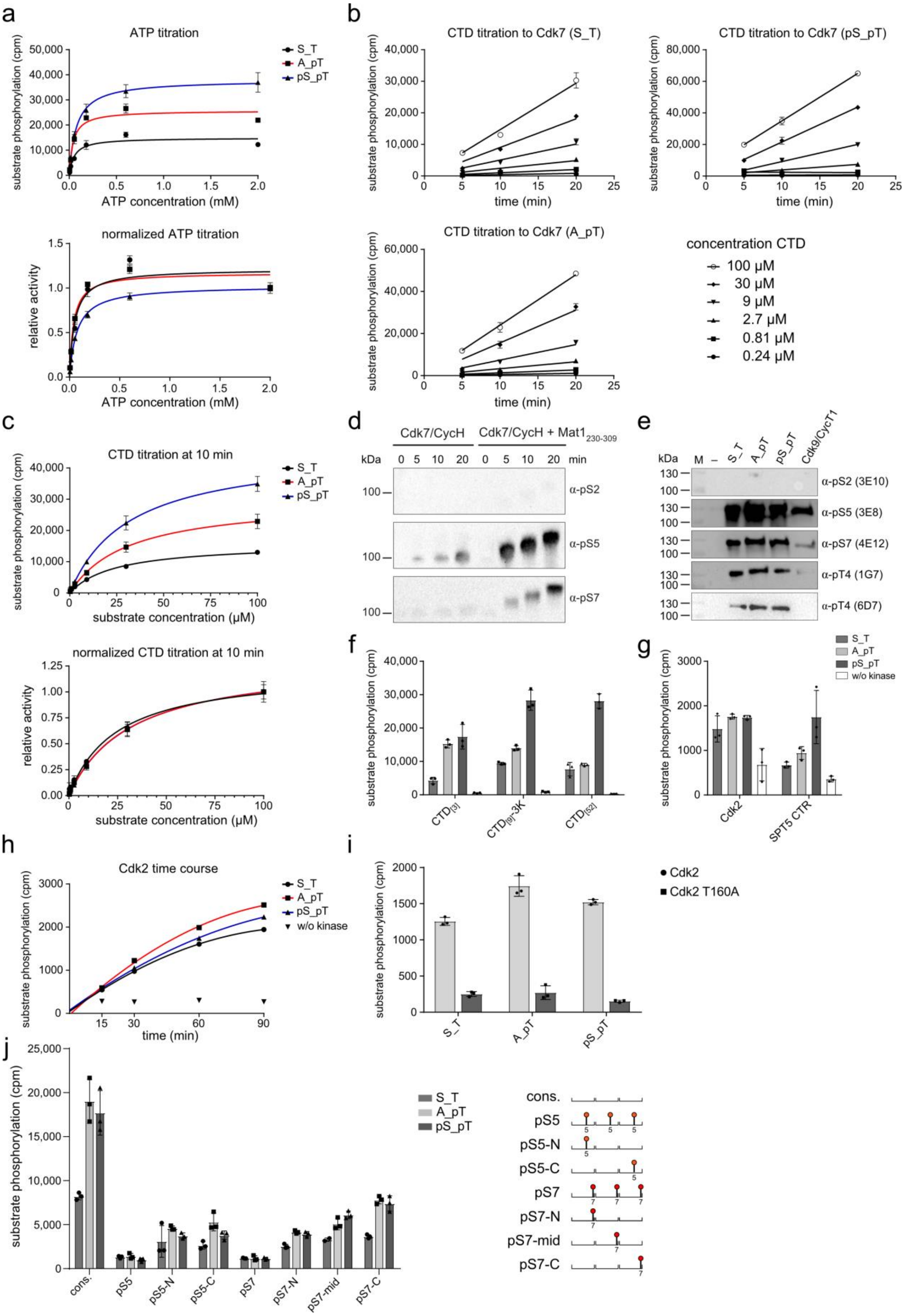
a
ATP titration
substrate phosphorylation (cpm)
ATP concentration (mM)
S_T
A_pT
pS_pT
normalized ATP titration
relative activity
b
CTD titration to Cdk7 (S_T)
CTD titration to Cdk7 (pS_pT)
CTD titration to Cdk7 (A_pT)
time (min)
concentration CTD
100 µM
30 µM
9 µM
2.7 µM
0.81 µM
0.24 µM
c
CTD titration at 10 min
substrate concentration (µM)
normalized CTD titration at 10 min
d
Cdk7/CycH
Cdk7/CycH + Mat1 230-309
kDa
min
α-pS2
α-pS5
α-pS7
e
M
Cdk9/CycT1
α-pS2 (3E10)
α-pS5 (3E8)
α-pS7 (4E12)
α-pT4 (1G7)
α-pT4 (6D7)
f
CTD[3]
CTD[9]3K
CTD[52]
g
w/o kinase
Cdk2
SPT5 CTR
h
Cdk2 time course
i
Cdk2
Cdk2 T160A
j
cons.
pS5
pS5-N
pS5-C
pS7
pS7-N
pS7-mid
pS7-C

Supplementary Fig. 5 | Substrate specificity within CTD is not altered by T-loop phosphorylation. **a**, Radiometric kinase assay. Kinase preparations (0.1 µM) were incubated with 50 µM GST-$CTD_{[9]}$ and indicated ATP concentration for 10 min at 30°C. Data are presented as absolute (upper panel) and normalized values (lower panel). K_M (ATP): S_T, 51.7 µM; A_pT, 40.3 µM; pS_pT, 75.5 µM. Data represent mean ± SD of triplicate measurements. K_M was determined by curve fitting according to the Michaelis-Menten equation. **b**, Radiometric kinase assay. Kinase preparations (0.1 µM) were incubated with indicated concentration of GST-$CTD_{[9]}$ and 1 mM ATP for indicated times at 30°C. Data represent mean ± SD of triplicate measurements. Lines were fitted by linear regression with the constraint X_0=0. **c**, K_M determination. CTD phosphorylation data after 10 min were extracted from **b** and used to determine K_M by applying Michaelis-Menten equation. Data are presented as absolute (upper panel) or normalized values (lower panel). Data represent mean ± SD of triplicate measurements. K_M(CTD): S_T, 24.3 µM; A_pT, 31.2 µM; pS_pT, 31.9 µM. **d**, Immunoblot analysis of site-specific CTD kinase activity of binary Cdk7/Cyclin H complex and Cdk7/Cyclin H incubated with MBP-$Mat1_{230-309}$ prior to activity assay. **e**, Immunoblot analysis of Cdk7 substrate site specificity compared to Cdk9/Cyclin T1 (P-TEFb). The clone number of the respective monoclonal antibody is displayed in brackets. **f,g**, Absolute values of the radiometric kinase assay shown in Fig.4d. Data were split to two graphs for better visualization. Data represent mean ± SD of triplicate measurements. **h**, Radiometric kinase assay. Time course analysis of Cdk2 phosphorylation by Cdk7. Cdk7 preparations (0.1 µM) were incubated with 15 µM GST-Cdk2 and 1 mM ATP for indicated times at 30°C. Data represent mean of duplicate measurements. Curves were fitted using a second order polynomial equation ($Y=A + B*X + C*X^2$). **i,** Radiometric kinase assay. The indicated Cdk7 preparations (0.1 µM) were used to phosphorylate 15 µM GST-Cdk2 or GST-Cdk2 T160A. Samples were incubated for 30 min at 30°C. Data are presented as mean ± SD of triplicate measurement. **j**, Radiometric activity assay. Peptides composed of three heptad repeats containing different pre-phosphorylations are shown in schematic diagram at right. Pre-phosphorylation was either continuous (pS5/pS7) or within a single repeat (pS5/7-C-terminal or middle or N-terminal); cons.: (consensus sequence with no pre-phosphorylation). Data represent mean ± SD of triplicate measurements. Source data are provided as a Source Data file.

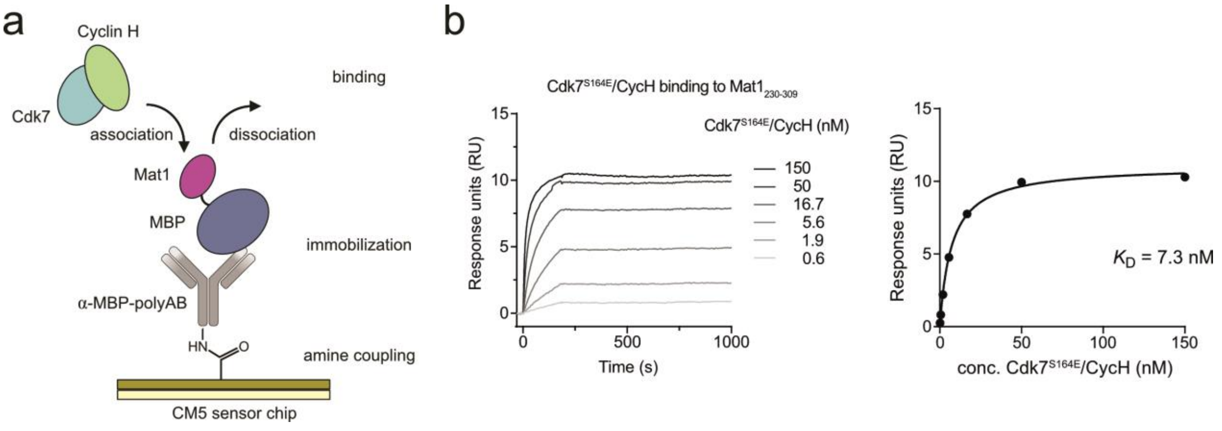

Supplementary Fig. 6 | SPR measurements. a, Experimental setup of the SPR measurements. MBP-Mat1 was immobilized as the ligand on an CM5 sensor chip by an α-MBP antibody and the Cdk7/Cyclin H complex was used as analyte in the flow cell. **b**, SPR analysis of Cdk7(S164E)/Cyclin H binding to Mat1. Source data are provided as a Source Data file.

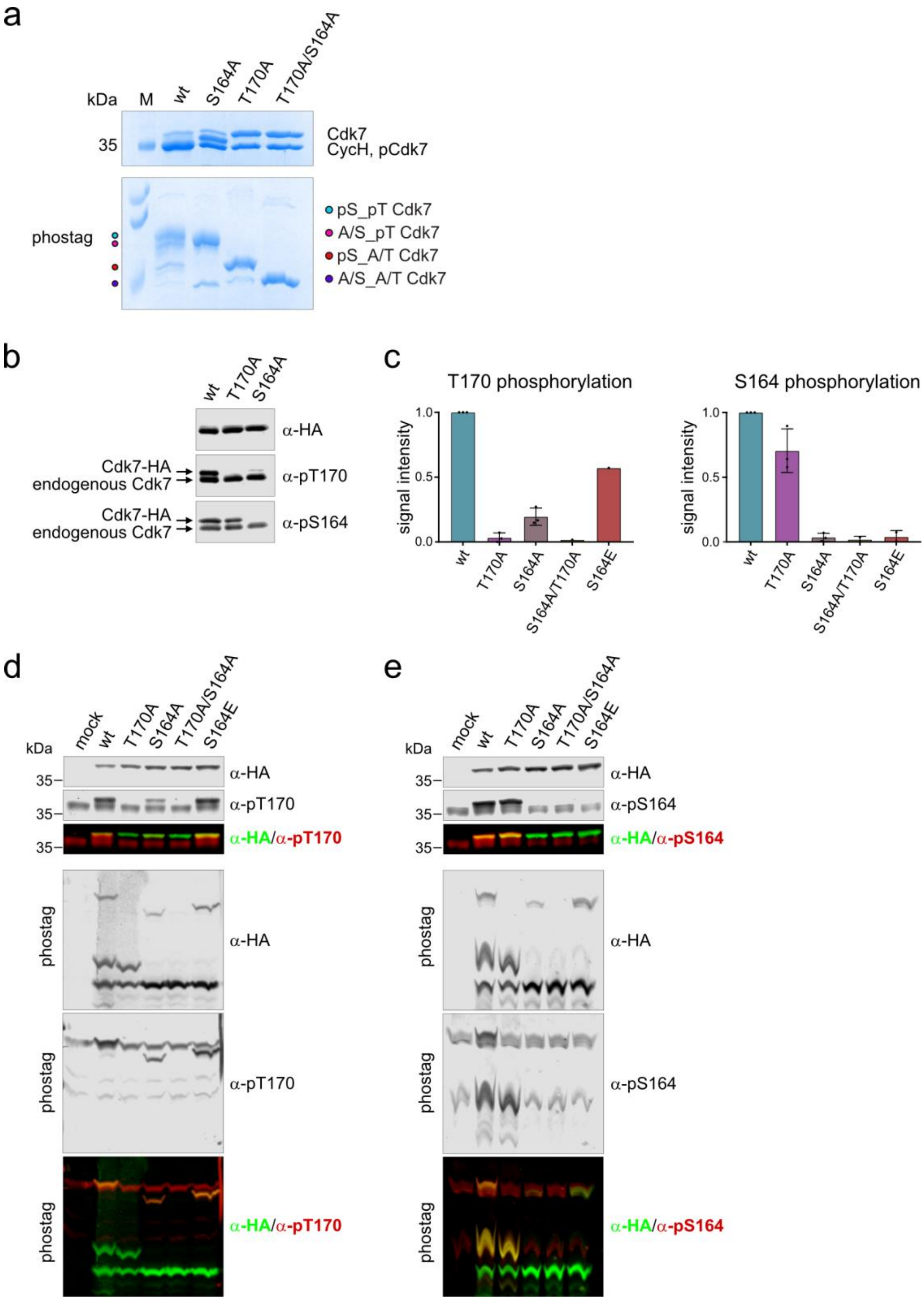

Supplementary Fig. 7 | Cdk7 T-loop phosphorylation in human cells. a, Conventional SDS-PAGE and phos-tag SDS PAGE analysis to discriminate Cdk7 phospho-isoforms. Recombinant GST-Cdk7/CycH samples shown in Fig. 3a were incubated with TEV protease to cleave off the GST-tag and 2 µg each resolved by SDS-PAGE and phos-tag SDS PAGE. **b**, Immunoblot analysis of Cdk7-HA transfected HCT116 cells. **c**, Quantification of immunoblot intensities for Cdk7 T170 and S164 phosphorylation. Phosphorylation signal intensity was normalized to the respective HA staining. The quotient of the Cdk7-HA wt sample of each blot was set to 1 for comparison of intensities. **d**,**e**, Conventional and phos-tag immunoblot analysis of Cdk7-HA transfected cells. Source data are provided as a Source Data file.

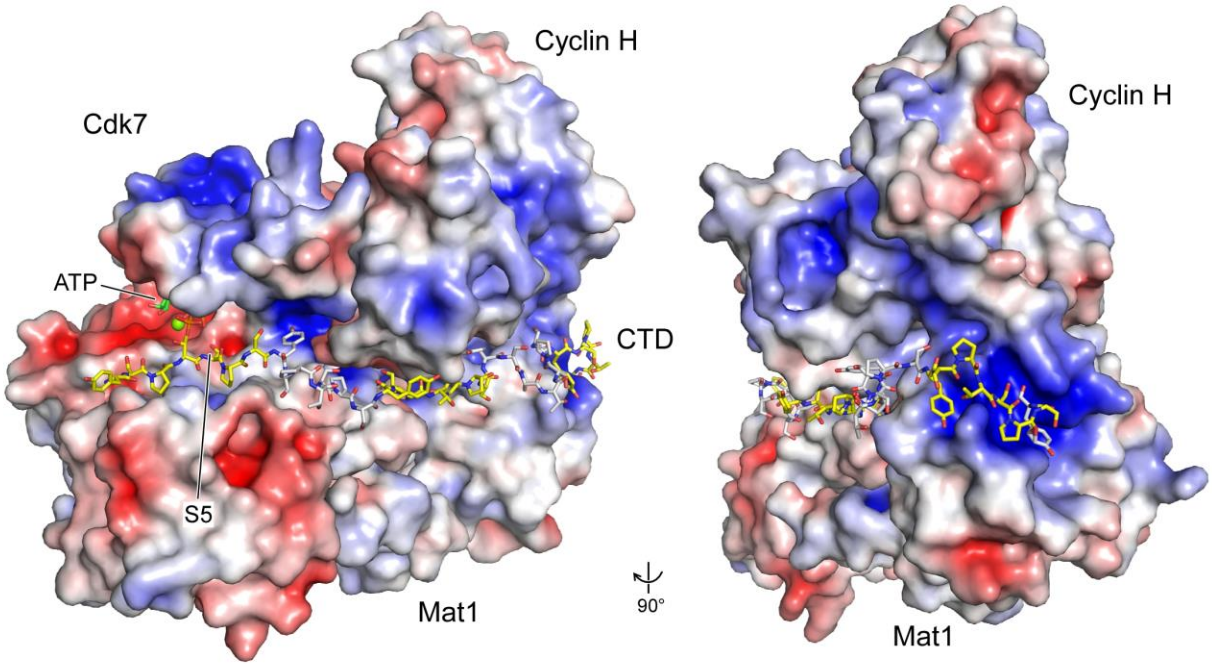

Supplementary Fig. 8 | Model of an RNAPII CTD substrate peptide of five repeats interacting with the Cdk7/Cyclin H/Mat1 complex. Electrostatic surface display of the Cdk7/Cyclin H/Mat1 complex structure determined here (8pyr) fused with residues 10-54, ATPγS and Mg^{2+} of Cdk7 from structure 6xbz [25] and superimposed with a substrate peptide at the catalytic site aligned to S5 phosphorylation that was modeled on the peptide PKTPKKAKKL from the Cdk2/CycA/substrate complex structure 3qhr [38]. The initial substrate model was extended at the N-terminus by residues Y_1S_2 and at the C-terminus by three heptad repeats, each repeat being alternately colored yellow and white. In this model, the fifth repeat was directed towards a basic patch on the second cyclin box of Cyclin H, which is formed by residues R197, R223, K253, R256 and K260.

Supplementary Table 1 Crystallographic data collection and refinement statistics.

	**Cdk7/Cyclin H/Mat1/VHH$_{RD7-04}$**
Data collection [a]	
Beam line	DESY P13
Wavelength [Å]	0.9763
Space group	P 1 21 1
Unit cell: a, b, c [Å]<br>α, β, γ [°]	119.86 77.87 121.87<br>90.0 119.31 90.0
Resolution range [Å]	47.49 - 2.15 (2.227 - 2.15)
Unique reflections	105,605 (9,833)
Multiplicity	10.2 (10.6)
Completeness (%)	98.97 (92.39)
Mean I/sigma(I)	7.79 (0.55)
R_{meas}	0.181 (3.309)
$CC_{1/2}$	0.998 (0.325)
Reflections used in refinement	105,571 (9,831)
Reflections used for R-free	2014 (192)
Refinement	
Model content	A: Cdk7 (50-311), B: Cyclin H (1-287), C: Mat1 (244-308),<br>D: VHH$_{RD7-04}$ (1-114)<br>E: Cdk7' (51-310), F: Cyclin H' (1-287). G: Mat1' (244-309),<br>H: VHH$_{RD7-04}$' (1-114)
# of atoms macromolecules	11,631
# of ligand atoms	45
# of solvent	350
Protein residues	1455
R_{work}	0.2027 (0.3409)
R_{free}	0.2379 (0.3631)
RMS deviations bonds [Å]	0.004
RMS deviations angles [°]	0.64
Ramachandran favored (%)	98.18
Ramachandran allowed (%)	1.68
Average B-factor	57.48
Macromolecules	57.54
ligands	65.00
solvent	54.56
PDB accession code	8pyr

[a] Values in parentheses are for the highest resolution shell.
R_{free}-value is equivalent to the R-value but is calculated for 5% of the reflections chosen at random and omitted from the refinement process.
