# Supplementary material for: Structural basis of Cdk7 activation by dual T-loop phosphorylation
Source: Nat Commun. 2024 Aug 3;15:6597. doi: 10.1038/s41467-024-50891-z (PMC11297931; doi:10.1038/s41467-024-50891-z)
Supplement: Supplementary file 3 — Reporting Summary [file 41467_2024_50891_MOESM3_ESM.pdf]

nature portfolio

Corresponding author(s): Matthias Geyer, Robert P. Fisher

Last updated by author(s): Jul 11, 2024

Reporting Summary

Nature Portfolio wishes to improve the reproducibility of the work that we publish. This form provides structure for consistency and transparency in reporting. For further information on Nature Portfolio policies, see our Editorial Policies and the Editorial Policy Checklist.

Statistics

For all statistical analyses, confirm that the following items are present in the figure legend, table legend, main text, or Methods section.

n/a	Confirmed	
[ ]	[x]	The exact sample size (*n*) for each experimental group/condition, given as a discrete number and unit of measurement
[ ]	[x]	A statement on whether measurements were taken from distinct samples or whether the same sample was measured repeatedly
[x]	[ ]	The statistical test(s) used AND whether they are one- or two-sided<br>*Only common tests should be described solely by name; describe more complex techniques in the Methods section.*
[x]	[ ]	A description of all covariates tested
[x]	[ ]	A description of any assumptions or corrections, such as tests of normality and adjustment for multiple comparisons
[ ]	[x]	A full description of the statistical parameters including central tendency (e.g. means) or other basic estimates (e.g. regression coefficient) AND variation (e.g. standard deviation) or associated estimates of uncertainty (e.g. confidence intervals)
[x]	[ ]	For null hypothesis testing, the test statistic (e.g. *F*, *t*, *r*) with confidence intervals, effect sizes, degrees of freedom and *P* value noted<br>*Give P values as exact values whenever suitable.*
[x]	[ ]	For Bayesian analysis, information on the choice of priors and Markov chain Monte Carlo settings
[x]	[ ]	For hierarchical and complex designs, identification of the appropriate level for tests and full reporting of outcomes
[x]	[ ]	Estimates of effect sizes (e.g. Cohen's *d*, Pearson's *r*), indicating how they were calculated

Our web collection on statistics for biologists contains articles on many of the points above.

Software and code

Policy information about availability of computer code

Data collection	X-ray crystallography data were collected at beamline P13 of the PETRA III synchrotron at the “Deutsches Elektronen-Synchrotron” (DESY) (Hamburg, Germany) at a wavelength of 0.9763 Ang.
Data analysis	X-ray crystallography data were analysed using PyMOL 2.0, Coot 0.9, PHENIX 1.19.1, XDS Version January 31, 2020 and MolProbity 4.5.1. SPR data were analysed with the Biacore Insight Evaluation Software (version 3.0.12.15655). Nano differential scanning fluorimetry (nanoDSF) data were analysed using the software PR.ThermControl version 2.1.5 (NanoTemper). SDS-gels and blots were analysed using ChemiDoc XRS+ system ImageLab (v.6.0.1) (BioRad) or Image Studio (v.5.5.4) (LI-COR) Graphs were plotted using GraphPad Prism (v.7).

For manuscripts utilizing custom algorithms or software that are central to the research but not yet described in published literature, software must be made available to editors and reviewers. We strongly encourage code deposition in a community repository (e.g. GitHub). See the Nature Portfolio guidelines for submitting code & software for further information.

Data

Policy information about availability of data

All manuscripts must include a data availability statement. This statement should provide the following information, where applicable:

- Accession codes, unique identifiers, or web links for publicly available datasets
- A description of any restrictions on data availability
- For clinical datasets or third party data, please ensure that the statement adheres to our policy

The authors declare that the data supporting the findings of this study are available within the paper and its supplementary information files. Source data are

provided with this paper. Structure coordinates and diffraction data of the human Cdk7–CycH–Mat1-VHHRD7-04 complex were deposited in the Protein Data Bank (http://www.pdb.org) under accession code 8pyr [http://doi.org/10.2210/pdb8PYR/pdb]. The coordinate data used in this study are available in the PDB database under accession codes 6xbz [http://doi.org/10.2210/pdb6XBZ/pdb], 3qhr [http://doi.org/10.2210/pdb3QHR/pdb], 1UA2 [http://doi.org/10.2210/pdb1UA2/pdb], 1N3X [http://doi.org/10.2210/pdb1N3X/pdb], and 8ORM [http://doi.org/10.2210/pdb8ORM/pdb].

Research involving human participants, their data, or biological material

Policy information about studies with human participants or human data. See also policy information about sex, gender (identity/presentation), and sexual orientation and race, ethnicity and racism.

Reporting on sex and gender	n/a
Reporting on race, ethnicity, or other socially relevant groupings	n/a
Population characteristics	n/a
Recruitment	n/a
Ethics oversight	n/a

Note that full information on the approval of the study protocol must also be provided in the manuscript.

Field-specific reporting

Please select the one below that is the best fit for your research. If you are not sure, read the appropriate sections before making your selection.

- [x] Life sciences
- [ ] Behavioural & social sciences
- [ ] Ecological, evolutionary & environmental sciences

For a reference copy of the document with all sections, see nature.com/documents/nr-reporting-summary-flat.pdf

Life sciences study design

All studies must disclose on these points even when the disclosure is negative.

Sample size	No sample size calculations were performed. No experients involving biological specimens were examined such that sample size does not apply. 18 crystals were screend to identify the optimal crystal for data collection. The number of crystals screened was random and was not limited by any experimental parameter. For surface plasmon resonance (SPR) experiments, optimal analyte concentrations were determined in initial screens, before binding affinities were determined in a single experiment.<br>Biochemical experiments were confirmed with multiple biological replicates as detailed in the Methods and Figure Legends.
Data exclusions	No data were excluded from analyses.
Replication	Crystals could be reproducibly grown and structures were determined at different resolutions. All other experiments were confirmed with multiple biological replicates as detailed in the Methods and Figure legends.
Randomization	No experiment involving animals or humans was performed in this study, therefore randomization is not applicable for this study.
Blinding	Blinding is not applicable for this study as no experiment involving humans or animals was performed.

Reporting for specific materials, systems and methods

We require information from authors about some types of materials, experimental systems and methods used in many studies. Here, indicate whether each material, system or method listed is relevant to your study. If you are not sure if a list item applies to your research, read the appropriate section before selecting a response.

Materials & experimental systems

n/a	Involved in the study	
[ ]	[x]	Antibodies
[ ]	[x]	Eukaryotic cell lines
[x]	[ ]	Palaeontology and archaeology
[x]	[ ]	Animals and other organisms
[x]	[ ]	Clinical data
[x]	[ ]	Dual use research of concern
[x]	[ ]	Plants

Methods

n/a	Involved in the study	
[x]	[ ]	ChIP-seq
[x]	[ ]	Flow cytometry
[x]	[ ]	MRI-based neuroimaging

Antibodies

Antibodies used	Primary antibodies: pSer2 CTD, rat monoclonal, clone3E10; pSer5 CTD, rat monoclonal, clone 3E8; pSer7 CTD, rat monoclonal, clone 4E12; pThr4 CTD, rat monoclonal, clone 1G7 (CTD antibodies were a kind gift from Dirk Eick, Munich) antibodies were validated in https://doi.org/10.1038/emboj.2012.123, https://doi.org/10.1126/science.1145977; pThr4 CTD, rat monoclonal, clone 6D7 (ActiveMotif Cat# 61362, RRID: AB_2750848; anti-Cdk7, mouse monoclonal, clone 31TF2-1F8 (Thermo Fisher Scientific Cat# MA3-001, RRID:AB_2608624); anti-Cdk7, mouse monoclonal, clone C4, (Santa Cruz Biotechnology Cat# sc-7344, RRID:AB_627243);phospho Cdk7 (pT170), rabbit polyclonal, (Affinity Biosciences #CPA5749); anti-phospho-Cdk7 (pT170), rabbit polyclonal, (Larochelle et al. 2012); anti-phospho-Cdk7 (pS164), rabbit polyclonal, (Thermo Fisher Scientific Cat# PA5-105583, RRID:AB_2817011); anti-GST, mouse monoclonal (Thermo Fisher Scientific Cat# 740007M, RRID:AB_3074177); anti-MBP, rabbit polyclonal, for immobilization on SPR sensor ship (Novus Biologicals, NBP2-22462).<br>Secondary antibodies: chicken anti-rat HRP (Santa Cruz Biotechnology Cat# sc-2956, RRID:AB_639257); goat anti-mouse IRDye 680RD, (LI-COR Biosciences Cat# 925-68070, RRID:AB_2651128); donkey anti-rabbit IRDye 800CW (LI-COR Biosciences Cat# 926-32213, RRID:AB_621848); goat anti-rat HRP, 1:100,000, (Cell Signaling Technology, Cat# 7077, RRID:AB_10694715
Validation	Commercial antibodies were used and tested according to the manufacturer's instructions. Antibodies from academic sources have been described and tested before (see references above). Specificity of Cdk7 phospho-antibodies was confirmed within this study by site specific mutation (Fig. 3a) and additional mass spectrometry (Fig. 1a and Supplementary Fig. 1c).

Eukaryotic cell lines

Policy information about cell lines and Sex and Gender in Research

Cell line source(s)	HCT116 (ATCC cat#CCL247)
Authentication	Cell lines were verified by manufacturer's website and cellular identity was regularly checked by morphology.
Mycoplasma contamination	Cell lines were tested mycoplasma negative through PCR.
Commonly misidentified lines (See ICLAC register)	No commonly misidentified cell lines were used.
