# Supplementary figures and images for: Structural basis of Cdk7 activation by dual T-loop phosphorylation
Source: Nat Commun. 2024 Aug 3;15:6597. doi: 10.1038/s41467-024-50891-z (PMC11297931; doi:10.1038/s41467-024-50891-z)

Figure 1a

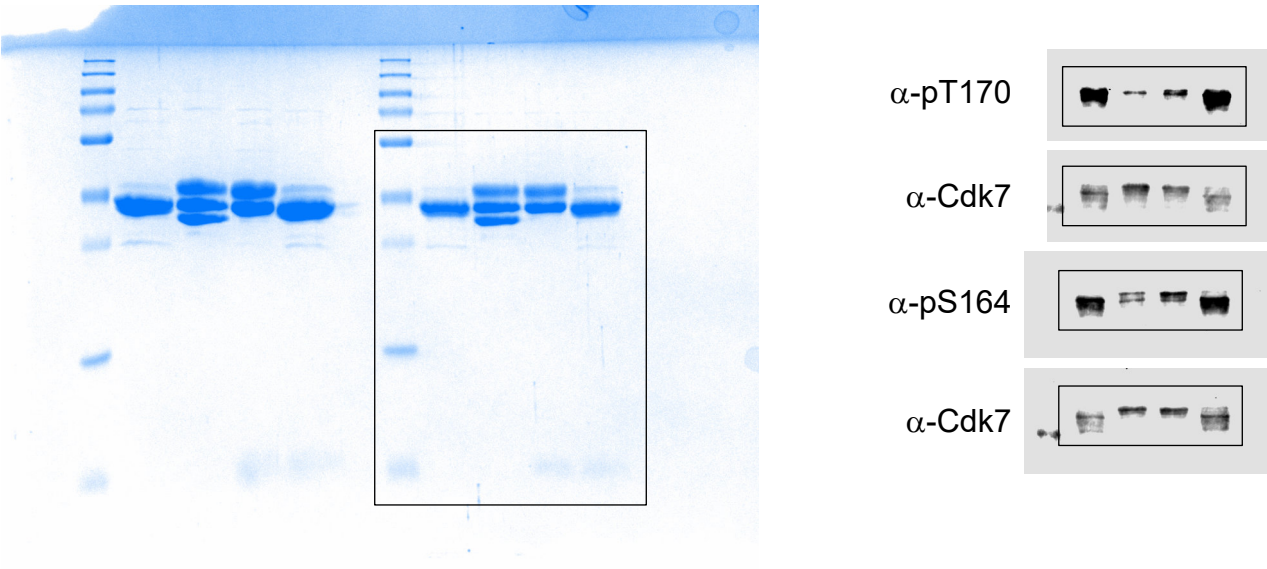

Supplement: Supplementary file 4 — Source data [file 41467_2024_50891_MOESM4_ESM.zip › Source data_Figure 1.pdf]

Figure 3a

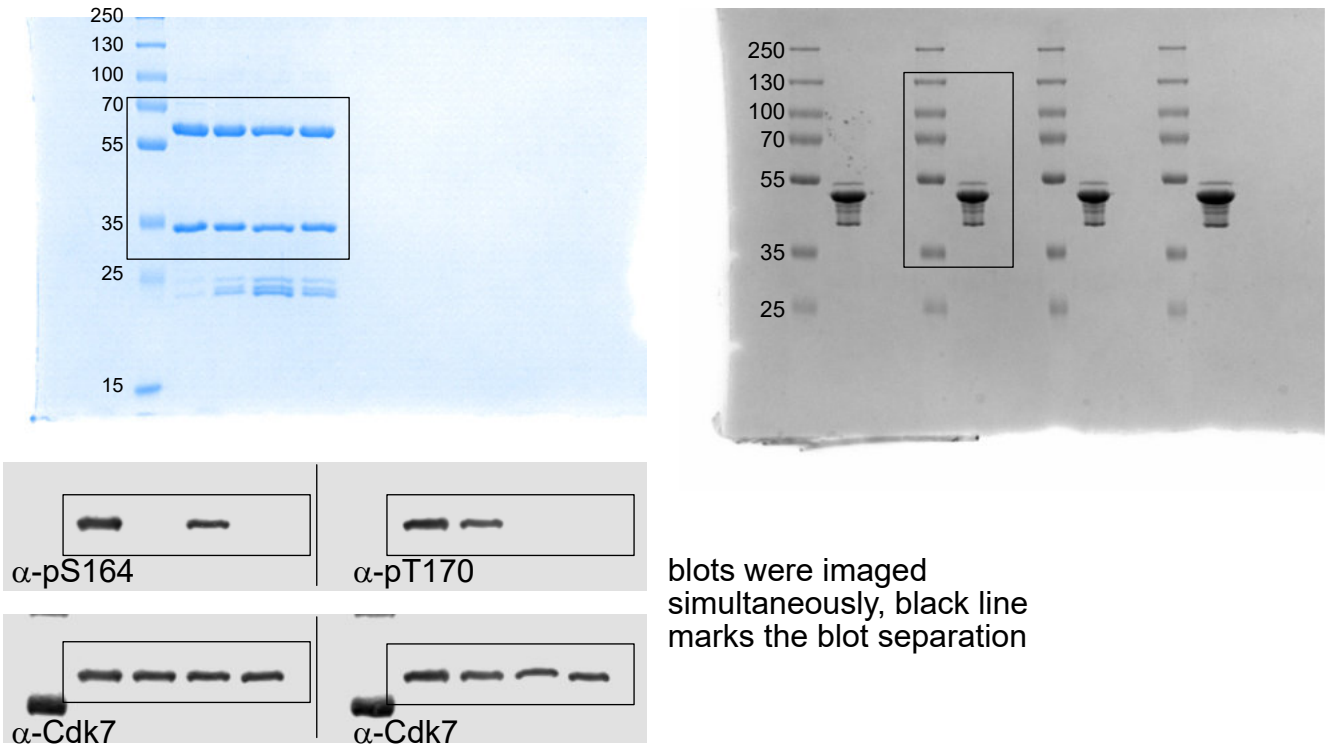

Figure 3c

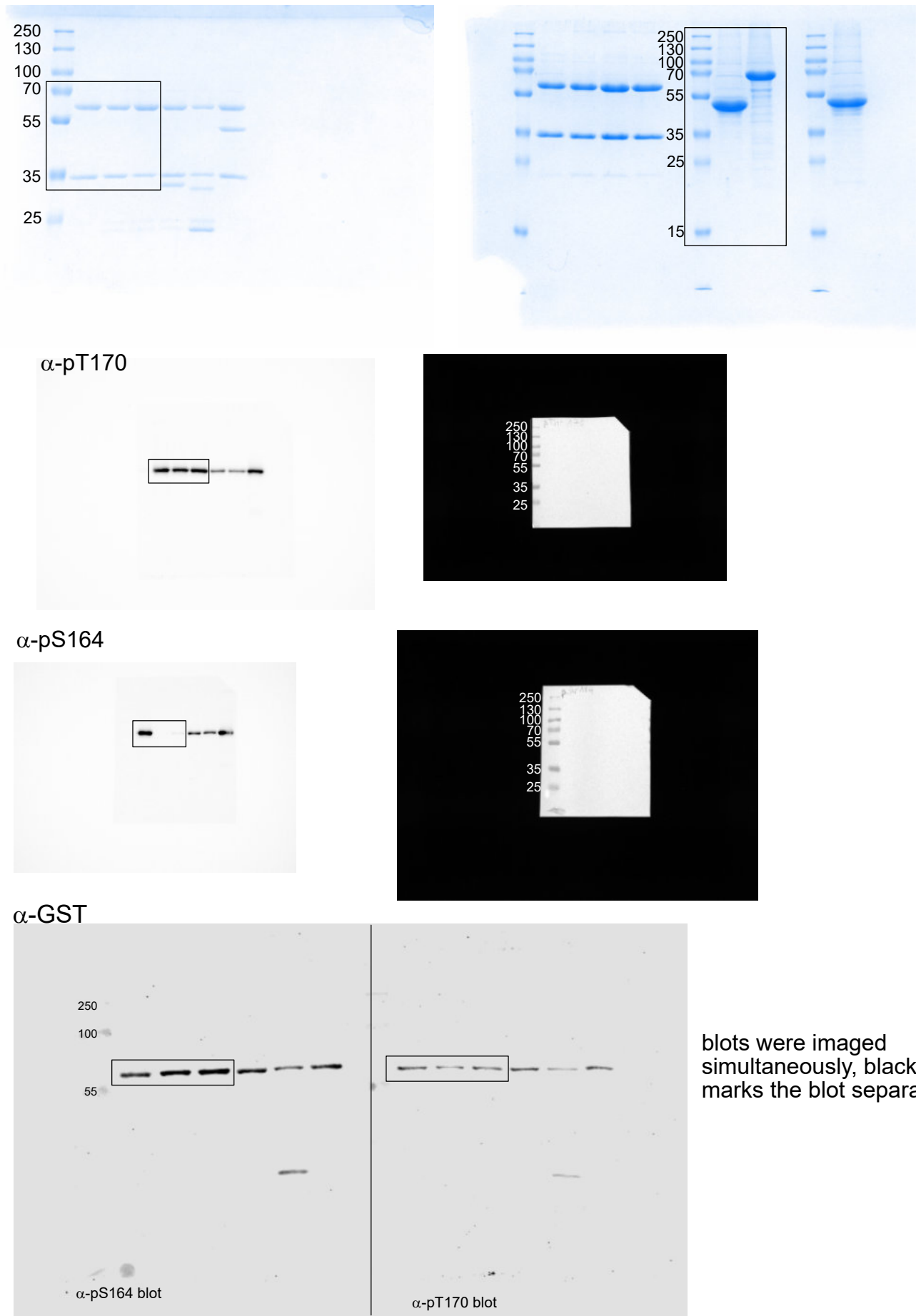

Supplement: Supplementary file 4 — Source data [file 41467_2024_50891_MOESM4_ESM.zip › Source Data_Figure 3.pdf]

Fig. 4c

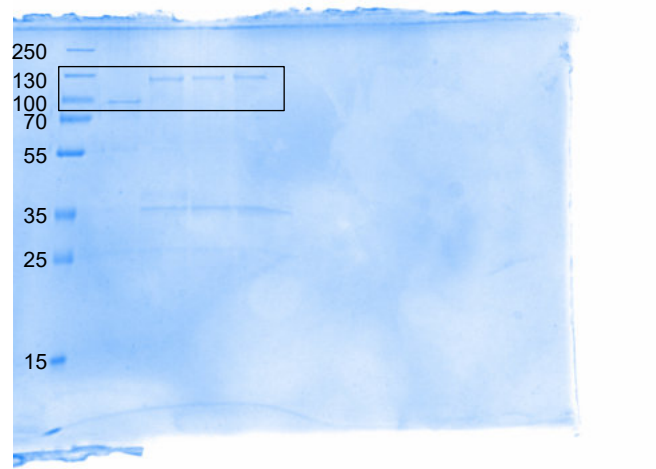

coomassie

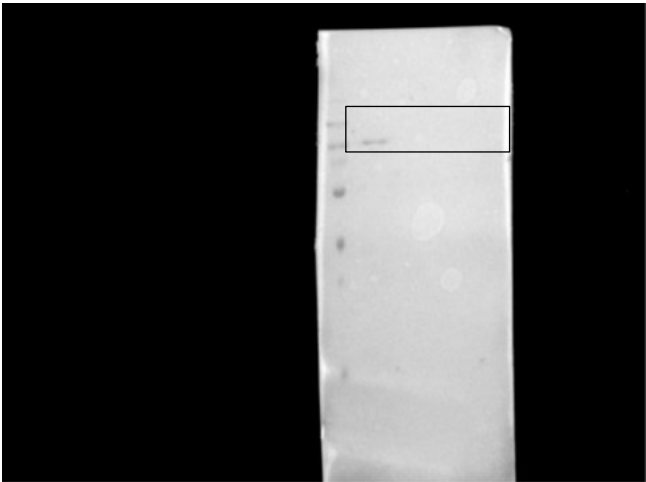

$\alpha$ -pS2

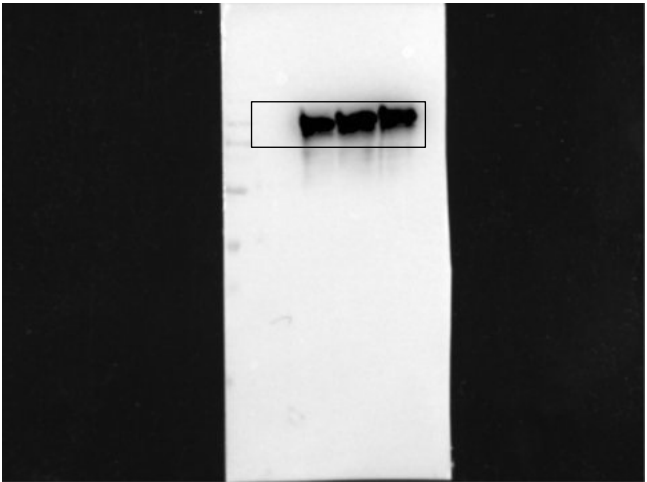

$\alpha$ -pS5

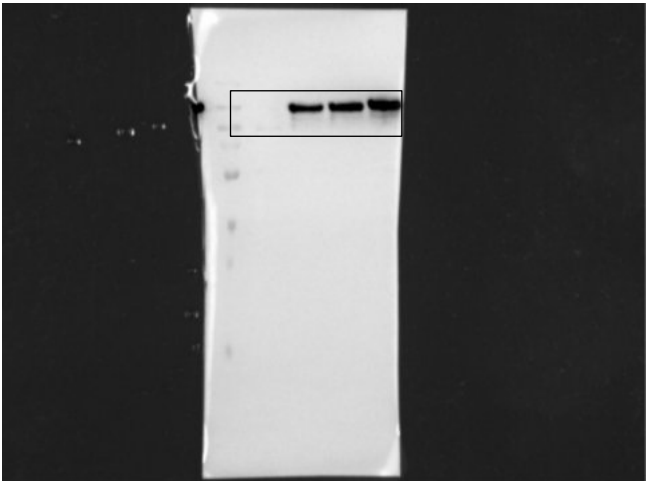

$\alpha$ -pS7

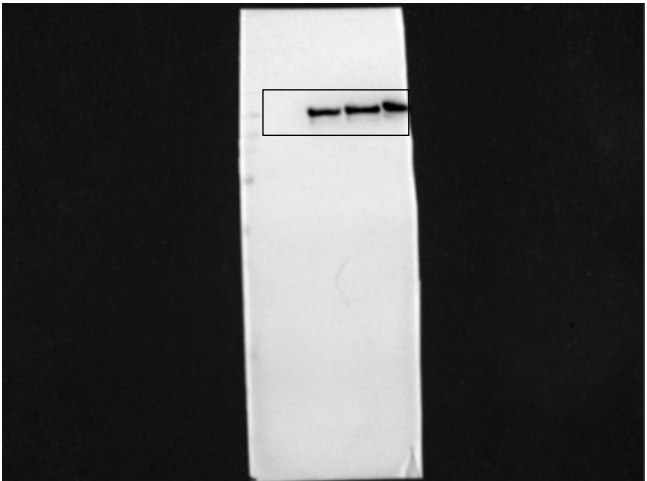

$\alpha$ -pT4

Supplement: Supplementary file 4 — Source data [file 41467_2024_50891_MOESM4_ESM.zip › Source Data_Figure 4.pdf]

Fig. 6a

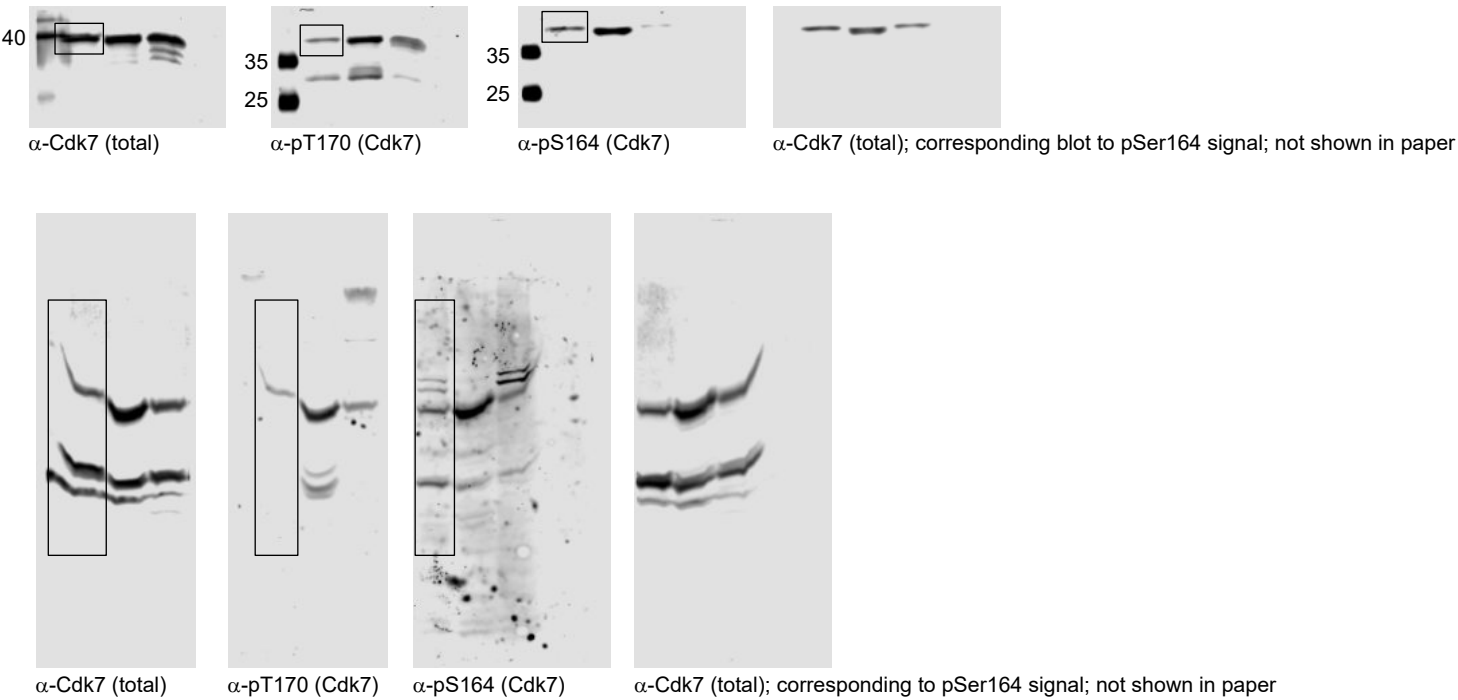

Fig. 6b

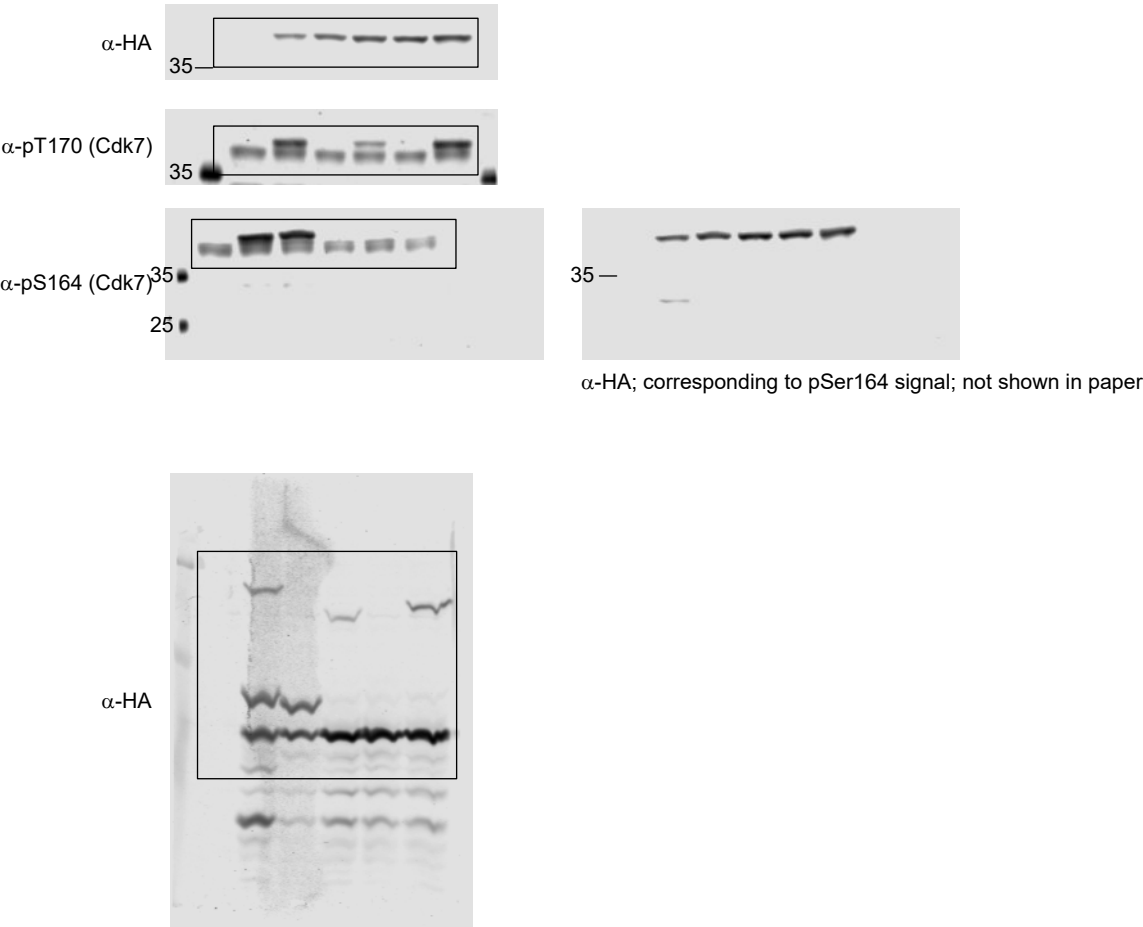

Supplement: Supplementary file 4 — Source data [file 41467_2024_50891_MOESM4_ESM.zip › Source Data_Figure 6.pdf]

Supplementary Fig. 1a

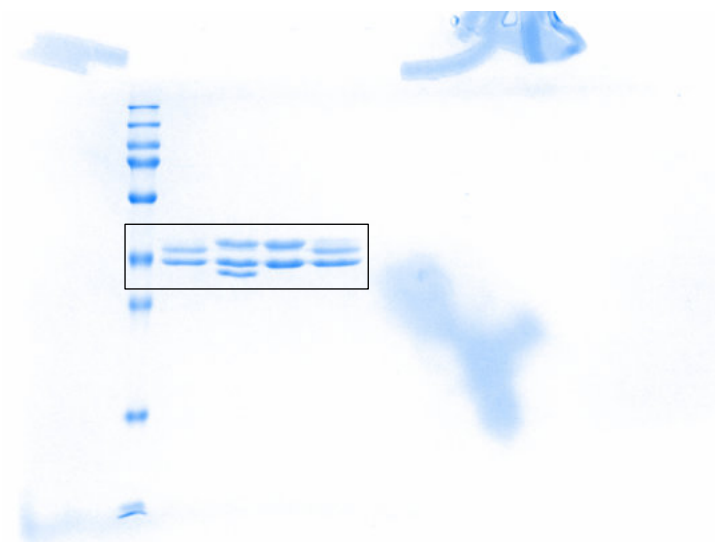

Supplementary Fig. 1c

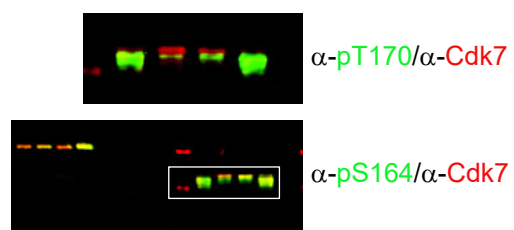

Supplement: Supplementary file 4 — Source data [file 41467_2024_50891_MOESM4_ESM.zip › Source Data_Supplementary Fig. 1.pdf]

Supplementary Fig. 2c

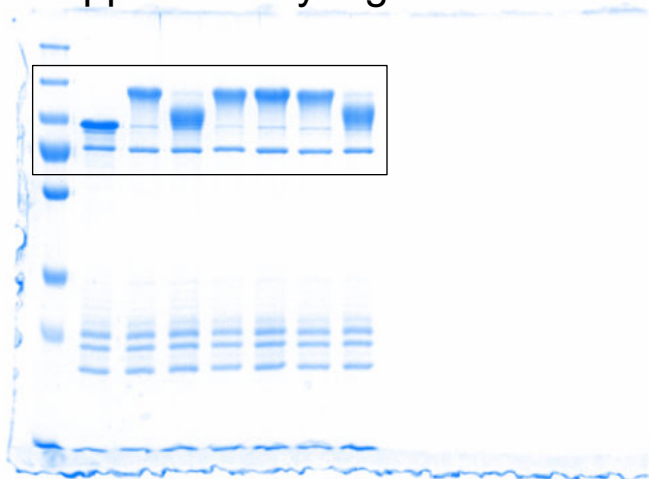

Supplement: Supplementary file 4 — Source data [file 41467_2024_50891_MOESM4_ESM.zip › Source Data_Supplementary Fig. 2.pdf]

Supplementary Fig.4a

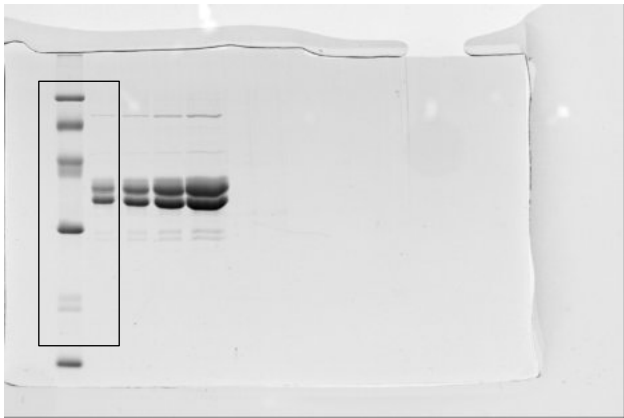

Supplementary Fig.4d

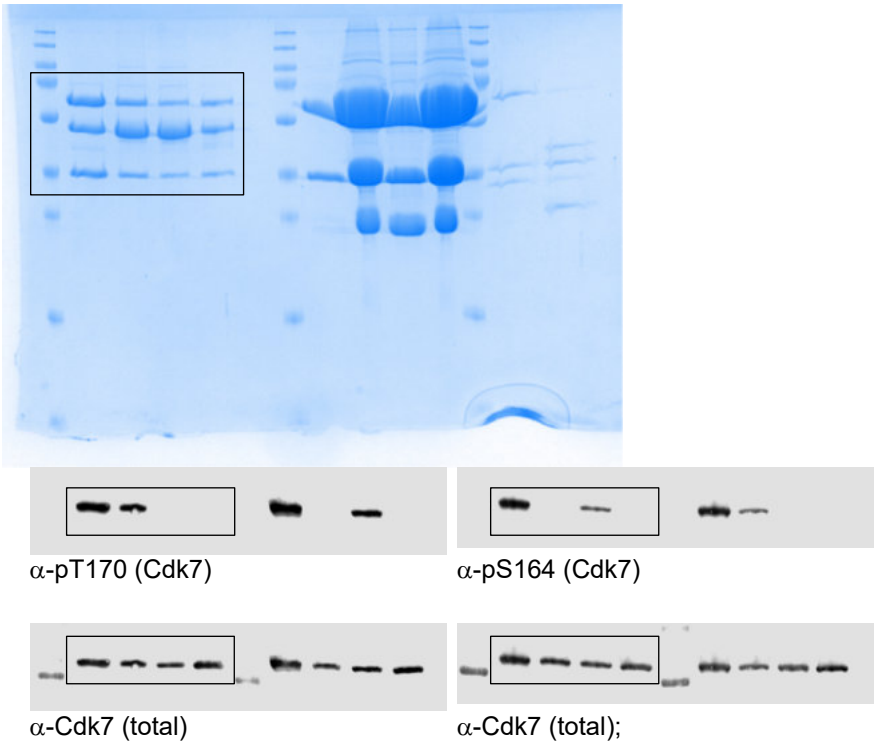

Supplementary Fig.4f

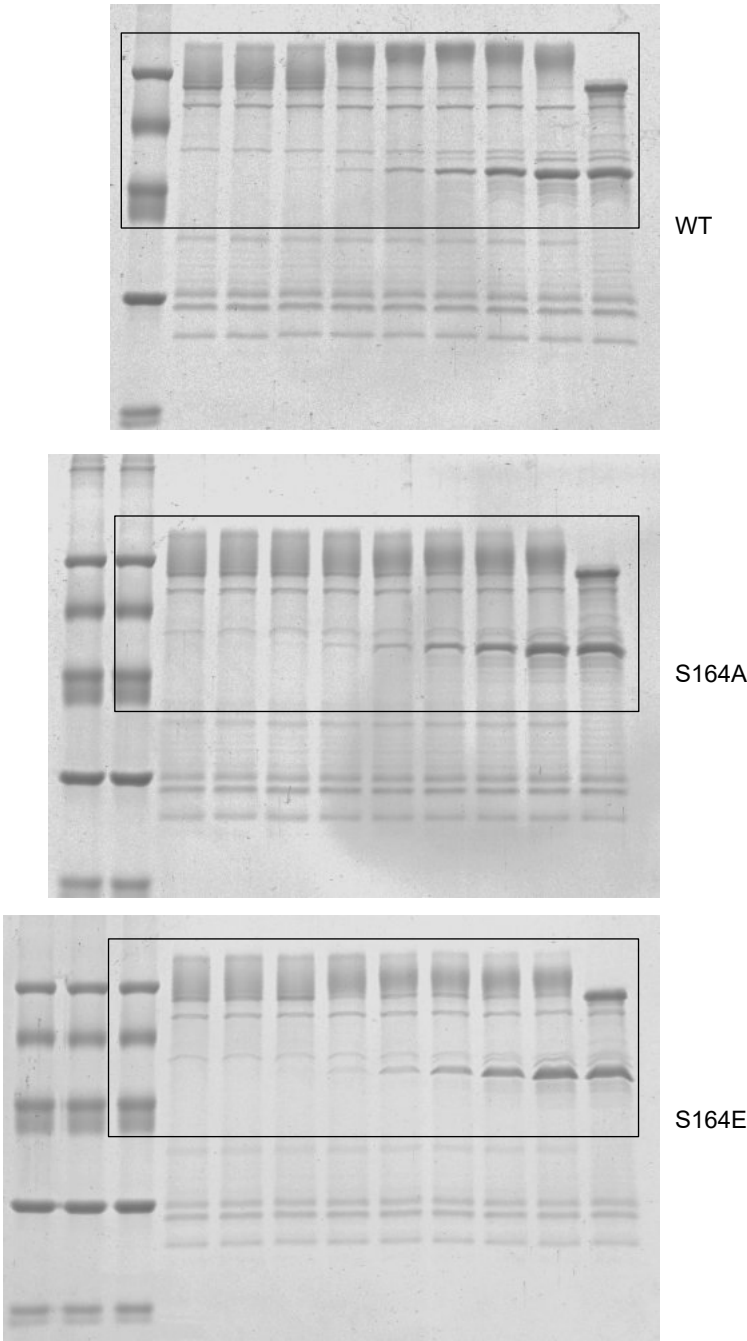

Supplementary Fig.4h

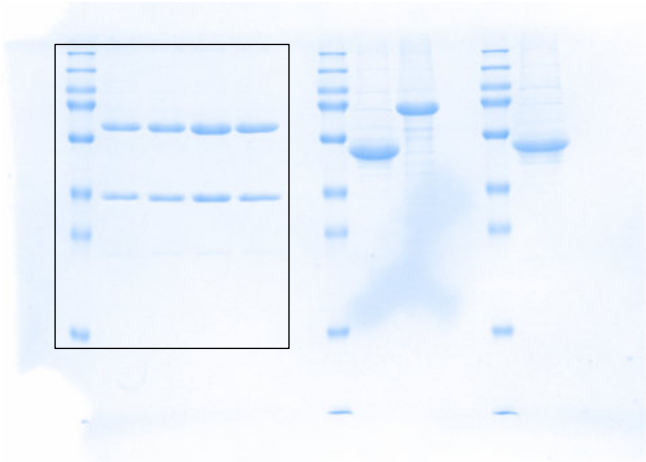

Supplement: Supplementary file 4 — Source data [file 41467_2024_50891_MOESM4_ESM.zip › Source Data_Supplementary Fig. 4.pdf]

Supplementary Fig. 4d

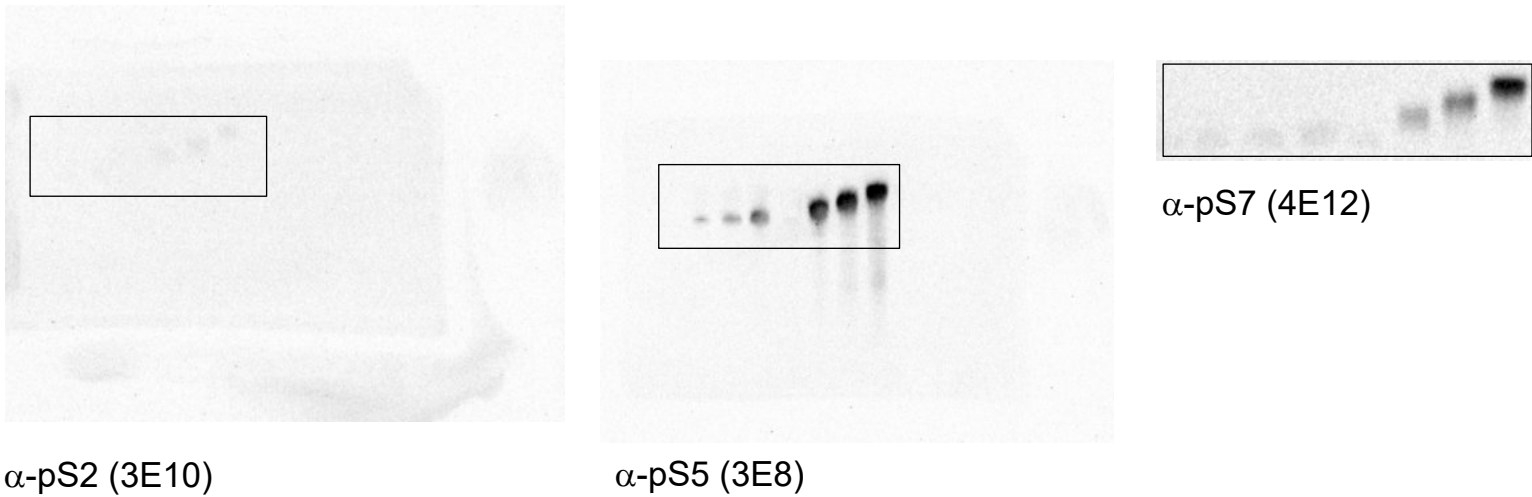

Supplementary Fig. 4e

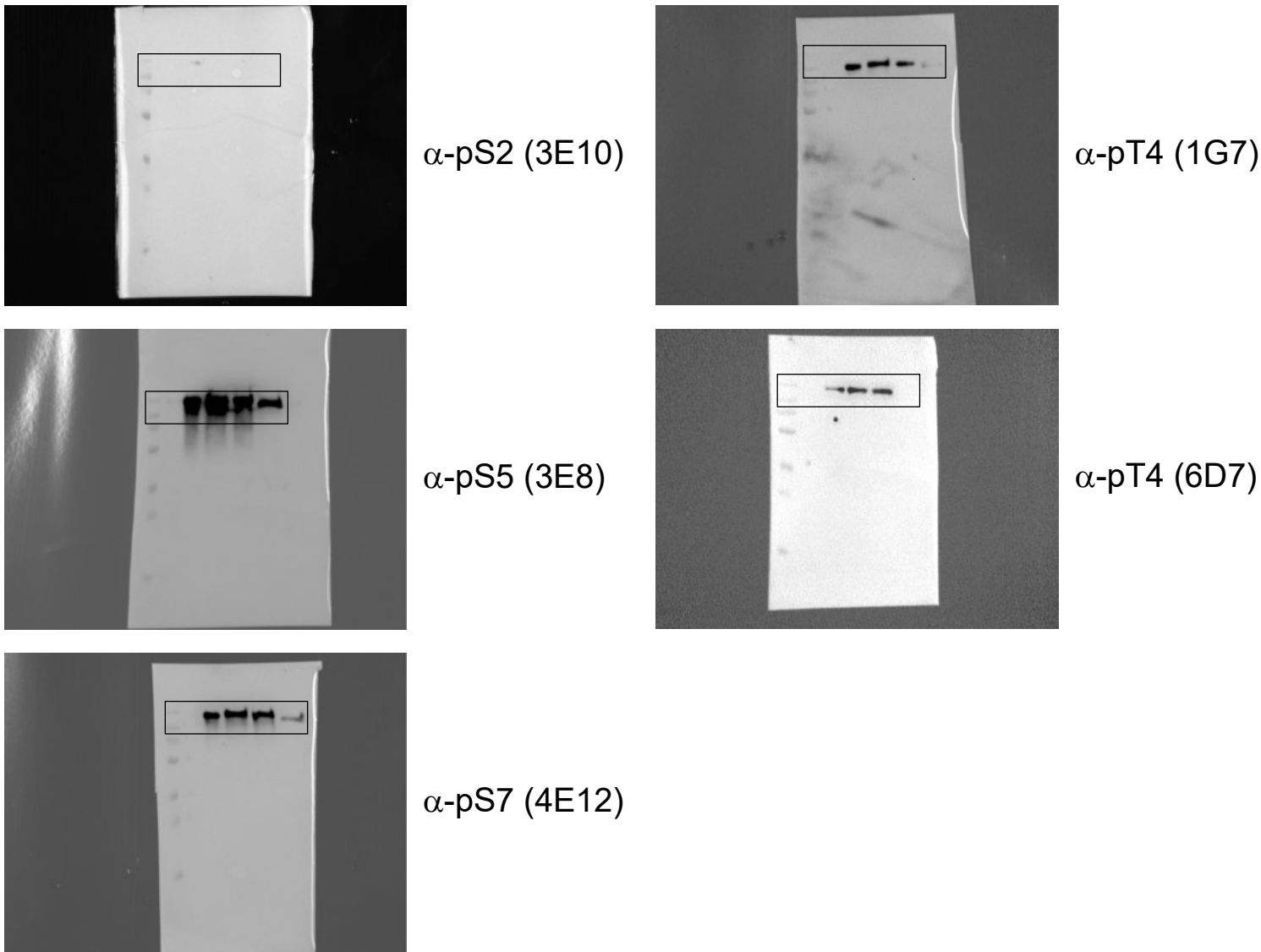

Supplement: Supplementary file 4 — Source data [file 41467_2024_50891_MOESM4_ESM.zip › Source Data_Supplementary Fig. 5.pdf]

Supplementary Fig. 7a

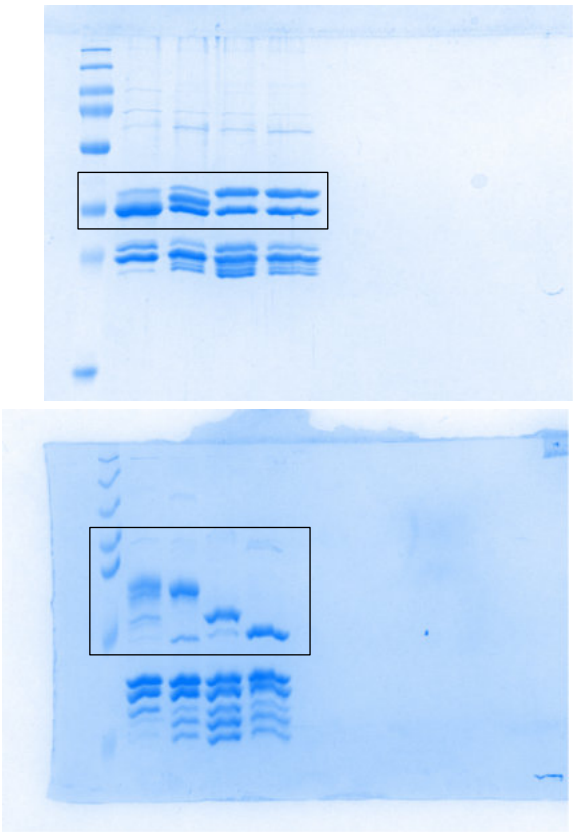

Supplementary Fig. 7b

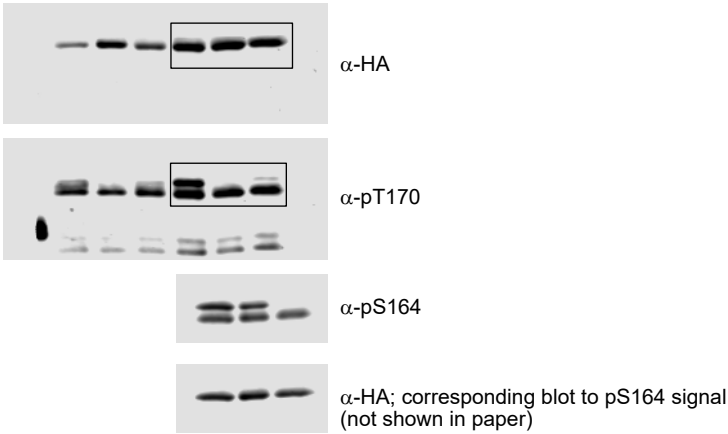

Supplementary Fig. 7d

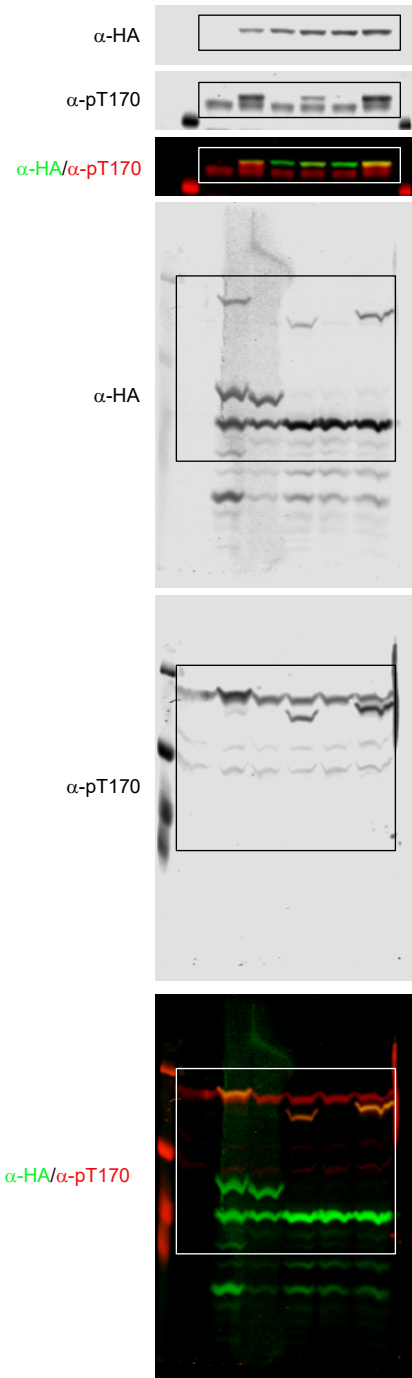

Supplementary Fig. 7e

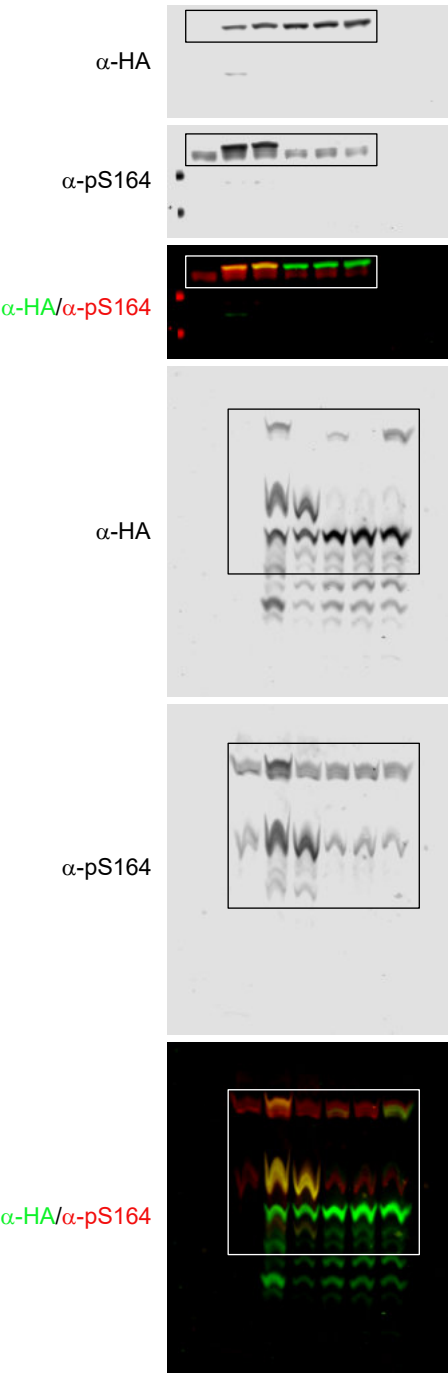

Supplement: Supplementary file 4 — Source data [file 41467_2024_50891_MOESM4_ESM.zip › Source Data_Supplementary Fig. 7.pdf]
